# Nutraceutical Value of Citrus Flavanones and Their Implications in Cardiovascular Disease

**DOI:** 10.3390/nu9050502

**Published:** 2017-05-16

**Authors:** Lara Testai, Vincenzo Calderone

**Affiliations:** 1 Department of Farmacia, University of Pisa, via Bonanno, 6 56120 Pisa, Italy; vincenzo.calderone@unipi.it; 2 Interdepartmental Center of Nutrafood, University of Pisa, via Del Borghetto, 80 56124 Pisa, Italy

**Keywords:** citrus flavonoids, cardiovascular benefit, nutraceutical value

## Abstract

Background- Cardiovascular diseases, including myocardial infarction, dyslipidaemia and coronary artery pathology, are a major cause of illness and death in Western countries. Therefore, identifying effective therapeutic approaches and their cellular signalling pathways is a challenging goal for medicine. In this regard, several epidemiological studies demonstrate a relationship between the intake of flavonoid-rich foods and the reduction of cardiovascular risk factors and mortality. In particular, flavonoids present in citrus fruits, such as oranges, bergamots, lemons and grapefruit (95% from flavanones), are emerging for their considerable nutraceutical value. Methods- In this review an examination of literature was performed while considering both epidemiological, clinical and pre-clinical evidence supporting the beneficial role of the flavanone class. We evaluated studies in which citrus fruit juices or single flavanone administration and cardiovascular risk factors were analysed; to identify these studies, an electronic search was conducted in PUBMED for papers fulfilling these criteria and written in English. Results- In addition to epidemiological evidence and clinical studies demonstrating that fruits in the *Citrus* genus significantly reduce the incidence of cardiovascular disease risk, pre-clinical investigations highlight cellular and subcellular targets that are responsible for these beneficial effects. There has been special attention on evaluating intracellular pathways involved in direct cardiovascular and cardiometabolic effects mediated by naringenin, hesperetin and eriodictyol or their glycosylated derivatives. Conclusions- Although some mechanisms of action remain unclear and bioavailability problems remain to be solved, the current evidence supports the use of a nutraceutical approach with citrus fruits to prevent and cure several aspects of cardiovascular disease.

## 1. Introduction

Cardiovascular diseases are a main cause of illness and death in Western countries, and cardiovascular drugs are the most commonly used medications. Therefore, cardiovascular diseases remain a therapeutic and sanitary issue, affecting the largest number of patients in the world. To alleviate the social and economic burden of cardiovascular diseases, recommendations have been proposed for health and lifestyle interventions targeting multiple risk factors. Prevention of risk factors is considered a primary approach for containing cardiovascular diseases [1].

The availability of nutraceuticals with a positive impact on cardiac function to reduce the incidence and lethality of cardiovascular diseases is a challenging topic [2,3].

In this regard, flavonoids are important constituents endowed with beneficial properties that humans can obtain through food, particularly through consuming fruit and vegetables. Some of these are characteristic of specific foods (i.e., genistein and daidzein), whereas others are widespread in several foods (i.e., quercetin and apigenin). Generally, flavonoids are distinct based on structural characteristics in the following six sub-classes: flavonols, flavones, isoflavones, flavanones, anthocyanins and flavanols (catechins and proanthocyanidins) [4].

In particular, the flavanone class is abundant in fruits and fruit juices of the *Citrus* genus; approximately 95% of flavonoids are represented by this sub-class [5], and these foods are the main source of flavanones. However, they are not unique because there are high levels in other foods, such as in tomatoes [6]. 

Citrus flavanones are glycosylated in vegetables; of note, a disaccaridic moiety is linked to the 7 position of aglycone and the aglycone type is characteristic of the fruit. Therefore, the same aglycone can be combined with several glycosides to give different flavanones; for example, the most representative flavanones in grapefruit are narirutin and naringin, those in orange fruit are hesperidin and narirutin, and that in lemon is eriocitrin. 

**Figure 1:**
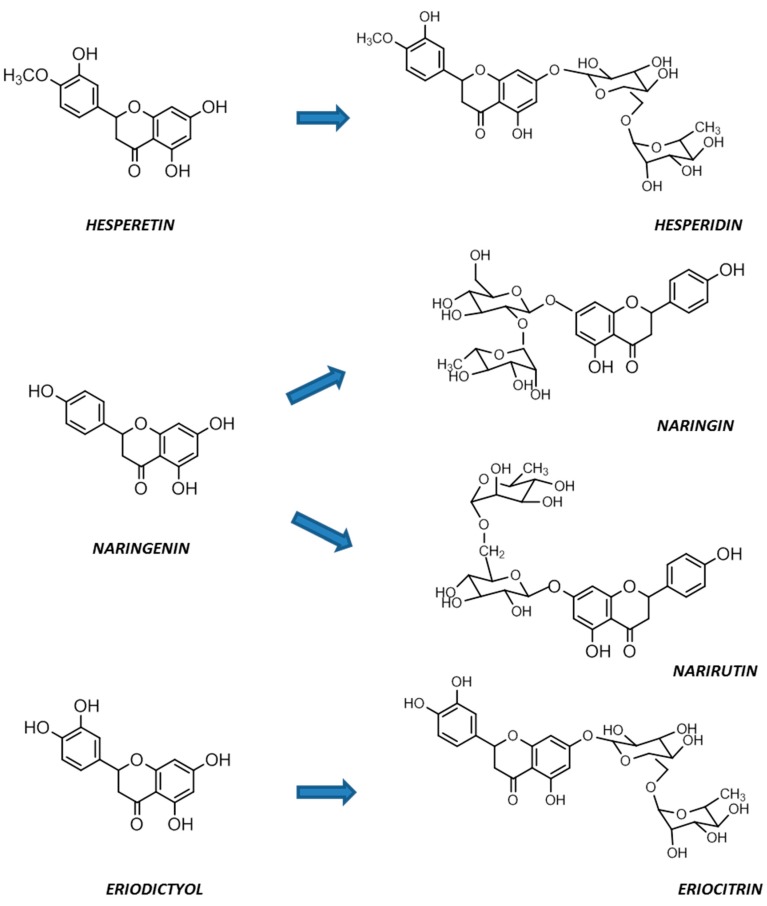
Chemical structures of citrus flavanones.

Of note, narirutin and naringin have the same aglycone, naringenin, and hesperidin is the glycoside of hesperetin, while eriocitrin contains the aglycone eriodictyol (Figure 1) [7]. 

These flavanones are not evenly distributed in the fruit; they are particularly present in the albedo and in the membranes separating cloves rather than in the pulp. Peterson and his colleagues report flavanone levels in the orange range between 35 and 147 mg/100 g, and naringin and narirutin in grapefruit are present in a range between 44 and 106 mg/100 g [5].

Because the albedo and membranous parts are usually discarded to prepare fruit juices, the actual level of flavanones is lower. Indeed, as reported by Tomas-Barbean and Clifford, the levels of hesperidin and narirutin in orange juice are between 13 and 77 mg/100 mg. Ross et al. quantified naringenin in grapefruit juice in the range of 17–76 mg/100 mL [8,9].

In Europe, orange or its fruit juice is the most commonly consumed citrus fruit; therefore, it is the principal fruit source of citrus flavanones [10]. Moreover, *O*-glycoside flavanones are present in all cultivars of orange, both red or pigmented and blond or non-pigmented; nevertheless, the number of flavanones is higher in red cultivars in which a high level of anthocyanins is also present, representing a peculiar feature [11]. 

## 2. Cardiovascular Benefits of Citrus Flavanones—Epidemiological, Clinical and Pre-Clinical Evidence

Epidemiological evidence and clinical and pre-clinical studies suggest that flavanones present in the *Citrus* genus positively influence cardio-metabolic parameters, preventing cardiovascular disease [12,13,14,15]. 

In particular, a recent epidemiological study performed a Nurses’ Health Study on approximately 70,000 women, highlighting an inverse correlation between flavanone intake and cerebral ischaemia risk, which is significant when considering women who consume high levels of flavanones (>63 mg/day) versus low levels (<13.7 mg/day) [16]. 

Another prospective study was performed in Finland on approximately 10,000 men and women, considering the correlation between the cardiovascular risk and flavonoid intake, revealing a 20% reduction in cerebrovascular diseases in those who consumed the highest levels of flavanones (4.7–26.8 mg aglycone/day) [17]. 

Similar results have been obtained in a Japanese cohort study conducted at JICHI Medical School. In Japan, citrus fruits represent 30% of the annual consumption of fruit and, in enrolled individuals, the incidence of cardiovascular diseases was evaluated during a period of approximately 11 years, confirming the inverse correlation between these [18]. 

Moreover, Wang and colleagues published a systematic review and meta-analysis of prospective cohort studies, which demonstrated that flavonoid consumption, especially of flavanones, was associated with a decreased risk of cardiovascular disease (*p* = 0.002) [19] 

Very recently, a meta-analysis of three randomized clinical trials, including 233 patients, demonstrated a correlation between grapefruit intake and a reduction in blood pressure. Although grapefruit intake does not significantly reduce body weight, it was responsible for a small, but significant, reduction in the systolic blood pressure and waist circumference in overweight and obese adults. The authors speculated that such beneficial effects can be related to naringin considering its great abundance in grapefruit [20].

Cassidy et al. reported approximately three prospective studies (Nurses’s Health Studies) in middle-aged and older US women and men in which the association between habitual intake of several flavonoid sub-classes and risk of incident hypertension was examined. This analysis confirmed that habitual flavonoid intake (principally from the consumption of flavanones present in grapefruits, oranges and citrus juices) is correlated with a reduced incidence of hypertension [21].

Another recognized cardiovascular risk factor is metabolic syndrome, a condition characterized by impaired glucose metabolism, dyslipidaemia, elevated blood pressure and abdominal obesity. Grosso et al. published a cohort study in 2016 on another 10,000 Polish subjects, demonstrating an interesting inverse association between polyphenols and metabolic syndrome, which is particularly evident in individuals with the biggest intake of these [22]. 

Therefore, evidence gathered thus far supports a preventive role of citrus fruits in addressing the main risk factors of cardiovascular diseases, including overweight, hypertension and hyperglycaemia; a deeper examination of specific cardio-vascular and cardio-metabolic parameters influenced by such a flavonoid sub-class has been performed below.

A schematic table of the epidemiological and clinical evidence supporting the cardiovascular benefits obtained with citrus flavanones is reported in Table 1.

### 2.1. Effects on Cardiovascular Parameters

#### 2.1.1. Reduction of Endothelial Dysfunction and Improvement of Vascular Function

The paradigm between hypertension and endothelial dysfunction has been widely demonstrated, as well as that between the reduction of endothelial integrity and atherosclerotic processes. Indeed, the vascular endothelium is a very active organ responsible for regulating vascular tone through the effects of locally synthesized mediators, especially nitric oxide (NO), endothelial NO synthase (eNOS), and superoxide, and its depletion is both a sign and cause of endothelial dysfunction resulting from reduced activity of eNOS and amplified production of reactive oxygen species. Then, the integrity and reactivity of endothelium must be ensured to prevent the progression of cardiovascular disease [35].

Clinical trials in the literature indicate a clear correlation between citrus flavanone intake, vasodilatation and reduction of endothelial dysfunction. In particular, Reshef and colleagues described a study on patients with mild hypertension (stage I) treated with sweetie fruit, which is a hybrid between grapefruit and pomelo that contains a high level of flavonoids from the *Citrus* genus (25% of naringin and 30% of narirutin). Two types of sweetie juices were obtained, one with a low flavonoid level (166 mg/L naringin and 64 mg/L narirutin) and one with a high flavonoid level (677 mg/L of naringin and 212 mg/L narirutin). After 5 weeks, a significant reduction in the diastolic pressure value was observed in the high flavonoid group (*p* = 0.04) [23]. In agreement with this study, a study on 4 weeks of treatment with 292 mg of hesperidin (corresponding to the levels present in 500 mL of orange juice) showed a reduction in the diastolic pressure by 4 mmHg in moderately overweight men (*p* = 0.02). Moreover, the intake of hesperidin supplement improved the post-prandial reactivity of the microvascular endothelium (*p* < 0.05), demonstrating that hesperidin can positively influence endothelial function. Based on these results, the authors encourage the consumption of citrus foods [24]. 

A further confirmation of these observations was demonstrated in a clinical trial performed on 25 patients with metabolic syndrome. After 3 weeks of treatment with 500 mg daily of capsules containing 98% of pure hesperidin, the expression of E-selectin, a biomarker of endothelial dysfunction, was significantly reduced. This clinical effect was accompanied by an improvement in endogenous NO production, inspiring the hypothesis that the vascular protection could be mediated by enhancement of endothelial function [25,26]. 

Of note, a primary risk factor for cardiovascular diseases in aged women is the post-menopausal condition, which is mainly due to dysfunction of the endothelium. Morand’s group reported a clinical trial enrolling 52 women after menopause who were asked to consume a bottle (340 mL) of a concentrated blond grapefruit juice (containing 105 mg of naringenin) or a iso-caloric and iso-energetic drink daily. After 6 months of treatment, volunteers consuming grapefruit juice had a significant change in their anthropometric parameters and vascular function, with improvement in arterial stiffness that was independent of blood pressure changes [36]. A positive impact of flavonoids on hypertension has also been shown in a prospective cohort on French middle-aged women [28]. In agreement with several previously described studies, improvement in vascular function and slowing of atherosclerosis progression have been reported in normotensive subjects (healthy volunteers and patients with type 2 diabetes or coronary artery disease) [37,38] and in overweight men [39].

#### 2.1.2. Lipid Level Reduction

Elevated levels of low-density lipoprotein cholesterol (LDL-C) and its deposition in the macrophages of arterial walls contribute to the main cause of atherosclerosis progression, which is a gateway for cardiovascular complications, including heart attacks, ischaemic stroke and coronary diseases [40]. Several large randomized clinical trials with statin-based and non-statin based therapies have demonstrated that a reduction in LDL-C levels reduced the cardiovascular risks [41]. 

Interestingly, naringenin promotes a decrease in LDL-C and triglycerides as well as inhibiting glucose uptake. On the other hand, it increases high-density lipoprotein (HDL-C) and ameliorates anti-oxidant defences, downregulating atherosclerosis-related genes [42].

Several pre-clinical studies have demonstrated the role of flavanones in atherosclerosis progression. In particular, a 0.1% naringin or 0.05% naringenin supplement given to rabbits with high cholesterol levels significantly decreased the expression levels of vascular cell adhesion molecule-1 (VCAM-1) and monocyte chemotactic protein-1 (MCP-1) after 8 weeks; at the same time, the hepatic cholesterol acyltransferase (ACAT) activity, the basis for reducing atherosclerotic plaques, appeared to decrease [43,44]. Similarly, another research group demonstrated that mice given high cholesterol levels plus naringin (0.02%, corresponding to a half grapefruit) had a significant reduction (41%) of atherosclerotic plaque compared to the high cholesterol level-alone group [45]. 

In terms of clinical evidence, the administration of capsules containing 400 mg/day of naringenin for two months promoted a drop in the LDL-C, cholesterol and ApoB levels and an increase in HDL-C levels in hypercholesterolemic, but not control, subjects, and there was an improvement in detoxifying enzymes [29]. 

Moreover, patients with metabolic syndrome given a supplement of hesperidin (500 mg/day) for 3 weeks had reduced cholesterol and ApoB levels [25]. In agreement, in a 2012 clinical study performed in Spain on patients with a diagnosis of metabolic syndrome, the glycaemic profile was unchanged after 4 and 6 months of citrus fruit juice treatment (300 mL daily), but the lipid profile improved, as observed by decrease in the cholesterol, LDL-C, and C-reactive peptide (a well-known inflammatory marker) levels (*p* < 0.05) [30]. 

Of the citrus fruits, bergamot fruit is worth noting because it is considered a promising nutraceutical approach for controlling hypercholesterolaemia. *Citrus bergamia* administered to rats with hyperlipidaemia (1 mL/rat/day) showed hepatic protection and a significant reduction in the cholesterol, triglycerides and LDL-C levels (−29%, −46% and −52%, respectively) with an approximately 28% increase in the HDL-C levels. These effects are the basis for anti-atherogenic activity and are responsible for cardiovascular disease prevention [46]. Additionally, the authors reported that bergamot can increase the excretion of sterols and bile acids. The mechanisms through which bergamot has beneficial effects are not completely clear, though it cannot only be due to citrus flavanones (naringenin, hesperetin and eriodictyol) [47]. Indeed, Di Donna et al. reported the presence of 3-hydroxy-3-methyl-glutaryl flavanones with a behaviour similar to simvastatin in a model of hypercholesterolaemic rats, speculating that these statin-like compounds could potentiate the hypocholesterolaemic effects [48]. 

However, beyond these encouraging data, a more nebulous scenario appears based on consideration for the citrus flavanone effects on hypercholesterolaemic patients. On the one hand, a study enrolling 25 volunteers with high LDL-C and cholesterol levels demonstrated that 4 weeks of feed with 600 mL/day of blond orange juice significantly reduced the plasma levels of oxidative stress markers and apo A levels [31]. In agreement, a recent 6-month prospective study showed that bergamia extract (containing 150 mg of flavonoids with 16% neoericitrin, 47% neohesperidin and 37% naringin) reduced the plasma levels of lipids and improved the lipoproteic profile in moderate hypercholesterolemic patients. Of note, such an ipolipidaemic effect was more evident in the group with higher cholesterol levels [32].

On the other hand, a clinical study with 500 mg of naringin plus 800 mg of hesperidin did not show a significant improvement in the lipid profile in moderate hypercholesterolaemia patients. Of note, the citrus flavanone doses in the study correspond to the 95th percentile of daily consumption in Western populations and were finalized to minimize the chance of not detecting a LDL-C lowering effect. This outcome suggests that citrus flavonoids have no effect on LDL-C in people, at least not when consumed in a capsule format [33]. Similar results have been reported in overweight patients consuming 292 mg of hesperidin and 47.5 mg of narirutin for 4 weeks [24]. A possible explanation for this discrepancy, according to some authors, could be caused by high inter-individual variability in the pharmacokinetic parameters beyond by the type formulation. Although pre-clinical results are clearer and encouraging, further clinical studies need to be performed. 

## 3. Putative Mechanisms of Action Responsible for the Cardiovascular Benefits of Citrus Flavanones 

### 3.1. Anti-Oxidant and Anti-Inflammatory Action

Oxidative stress and inflammation are pathologic processes that contribute to atherosclerotic progression and the evolution of cardiovascular diseases. In healthy subjects, citrus flavanones do not have significant anti-oxidant effects, suggesting that their anti-oxidant potency is negligible in normal conditions. In hypercholesterolaemic subjects, Jung et al. demonstrated that naringin, administered for 8 weeks at a dose of 400 mg/day, significantly increased the SOD and catalase levels [29]. This result suggests that flavanones of the *Citrus* genus could have an important impact on the improvement of endogenous anti-oxidant defences in dyslipidaemia. Very recently, hesperidin has been demonstrated to elevate anti-oxidant defences through increasing Nrf2 expression, suggesting an anti-ageing effect of this flavonoid on senescent rat hearts [34]. Similar results emerged from an in vitro study on H9c2 cells in which naringenin significantly reduced the production of beta-galactosidase, a typical marker of senescence, after doxorubicin treatment [49].

Several in vivo studies showed that flavanones can reduce chemokines as well as inflammatory and adhesion molecules, whose expression is tightly regulated by the pro-inflammatory factor NF-kB. This anti-inflammatory action accounts for anti-atherogenesis at the endothelium, smooth muscle cell and monocyte/macrophage levels [50,51,52]. 

Five hundred milligrams of hesperidin can help reduce the plasma values of inflammatory factors and genetic expression of proteins involved in cell proliferation, chemotaxis and platelet adhesion [23]. Moreover, a very recent in vitro study on human endothelial cells, showed that hesperetin and its main metabolites inhibited TNF-α-induced cell migration [53]. On the other hand, 5 µM naringenin decreased the production of a pro-inflammatory eicosanoid, PGE2, and reduced the expression of the COX2 enzyme, while higher concentrations (30–100 µM) inhibited NFkB activation [54,55,56]. A further central inflammatory target is represented by matrix metallopeptidases; MMP9 is particularly involved in atherosclerotic lesions, and one study indicated that naringenin and naringin reduced MMP9 expression, reducing smooth muscle cell migration. Such an action could be at least partly related to the suppression of NF-kB activation [57].

### 3.2. Vasodilator Activity

Vasoactive properties of the main flavonoids of the *Citrus* genus, naringenin and hesperetin, have been widely described; particularly, Rizza et al. demonstrated that hesperetin induces vasodilatation through endothelial production of nitric oxide (NO) and its derivative, glycosyl-hesperidin, which was administered for 8 weeks to spontaneously hypertensive rats, was able to reduce pressure parameters 3% in addition to improving the endothelial response [25,58,59,60]. Moreover, on isolated coronary arteries of rodents, hesperetin caused vasodilatation by activating voltage-operated calcium channels and potassium currents [61]. 

With respect to naringenin-mediated vasorelaxing effects, they are probably linked to opening a calcium-activated potassium channel (BKCa) located on the sarcolemmatic membrane of smooth muscle cells, as demonstrated by Saponara and colleagues [62,63].

Finally, a unique paper reported the vasorelaxing property of eriodictyol, a flavanone typical of lemon; nevertheless, the authors reported a concentration-dependent reduction in the vascular tone in the rat aortic rings without elucidating the mechanism of action. More recently, in vitro protective effects on endothelial cells have also been demonstrated [64,65].

### 3.3. Anti-Ischaemic Activity

Myocardial infarction represents the main and often lethal manifestation of cardiovascular risk such that agents able to prevent it are useful for containing the damage.

Several pre-clinical studies have demonstrated the cardioprotection conferred by citrus flavanones. In ex vivo and in vivo myocardial ischaemia–reperfusion (I/R) models, naringenin could confer cardioprotection, and this action seemed to be mediated through activation of BKCa channels expressed on the inner mitochondrial membrane. This channel is structurally similar to that expressed on the sarcolemma and is involved in vasodilatation that, at the mitochondrial level, plays a crucial role in I/R events. Indeed, naringenin promoted reduced mitochondrial calcium uptake and mild mitochondrial depolarization as well as restricting the probability of mitochondrial permeability transition pore (MPTP) formation and apoptotic death of myocardiocytes [66,67,68].

On the other hand, hesperetin can have anti-apoptotic effects on cardiomyoblasts through the mitochondrial JNK/Bax pathway [69]. 

### 3.4. Glucose Tolerance

A supplement for 4 or more weeks with flavanones reduces glycaemia and insulinaemia in diabetic or insulin-resistant animals fed a high fat diet; moreover, glucose tolerance was improved. The insulin-like property of naringenin has been demonstrated, and it has added to the in vitro evidence that demonstrates the ability of naringin and hesperidin to reduce the PPAR-γ expression and glucokinase activity, a key enzyme involved in the glucose use [70,71,72]. In this context, a poly-methoxy flavone abundant in mandarin, tangeretin, should be mentioned. In diabetic rats, tangeretin markedly reduced the plasmatic glucose levels, while it also increased the insulin secretion, enhancing complex glucose metabolism [73].

## 4. Pharmacokinetic Profile of Citrus Flavanones

A significant problem with citrus flavonoids is their low bioavailability, restricting their efficacy to the point of having to enrich the fruit juice with citrus flavanones or their analogues, which are enzymatically more stable. Usually, the peak plasma concentration is reached 6 h after consumption with a µM concentration and relative differences among several flavonoid types [74]. 

Their metabolism concerns conjugation at the intestinal and hepatic levels, leading to two types of metabolites, glucuronide- and sulfate-conjugated. Principal excretion occurs through urine with a peak between 6 and 12 hours after intake [75]. 

Another factor that could influence the bioavailability of citrus flavanones is their solubility in fruit juice, and the preparation technique, homemade or industrial, is very important. Indeed, an analytical evaluation revealed that the qualitative composition is not changed, but quantitative analysis highlights significant differences [76]. However, the matrix factor is not the most critical. The main factor responsible for the high inter-individual variability seems to be the colon bacteria microflora [77], which is essential for flavonoid metabolism. Indeed, the consumption of fruit juice guarantees intake of glycosylated flavanones, but they are pro-drugs that require bio-activation via the hydrolysis of the glycoside portion to release the aglycone that is then responsible for pharmacological activity. Of note, an innovative view to improve the bioavailability of flavonoids and, more generally, of polyphenols is represented by the evaluation of human intestinal microbiota. Indeed, it has been demonstrated that the intestinal microbiota controls the bio-activation of flavonoids and regulates their catabolism [78]. On the other hand, other authors have noted that polyphenolic components could act as prebiotics and influence the growth of intestinal bacteria [79]. Further knowledge of the role of microbiota in metabolic diseases, as well as in cardiovascular diseases, and of factors that modulate microbiota will allow us to understand the true therapeutic benefits of various diet components and suggest an appropriate diet that optimizes these beneficial effects [80].

## 5. Conclusions

In conclusion, the mechanisms responsible for the beneficial effects of citrus flavanones on the cardiovascular system are multiple and remain somewhat unclear, although, in general, the available clinical and pre-clinical studies suggest a positive correlation between their intake and a significant reduction in the cardiovascular risk factors. However, such evidence is satisfactory to confer citrus fruits with an interesting nutraceutical value in the context of the spread of cardiovascular disease in Western countries and their heavy impact on the quality of life of patients.

Indeed, to date, cardiovascular drugs represent the most commonly used category in the world, and, although there are large-scale pharmacological treatments, cardiovascular diseases are the most widespread, consuming a high level of therapeutic resources and affecting health significantly. Furthermore, their prevalence is expected to rise, particularly in Western countries, because of obesity and the ageing population. 

A nutraceutical approach, such as with citrus fruits, aimed at preventing and curing several aspects of cardiovascular diseases could be very useful. 

## Figures and Tables

**Table 1:** Epidemiological evidence, clinical trials or meta-analysis in which beneficial effects of citrus flavanones or citrus fruits have been studied.

Type and Duration of Study	Number of Subjects Enrolled	Dietary Intervention	Outcomes	Reference
Epidemiological study “Nurses’s Healthy Study”	70,000 women	Flavonoid intake (>63 mg/day)	Reduction of cerebral ischaemia risk	[16]
Finnish prospective study	10,000 men and women	Flavanone intake (4.7–26.8 mg aglycone/day)	Significant reduction of cerebrovascular diseases (20%)	[17]
Japanese cohort study	12,500 men and women	Habitual citrus fruit consumption	Significant reduction of cardiovascular disease incidence (30%)	[18]
Meta-analysis of three prospective cohort studies	250 participants	Naringenin contained in grapefruit	Significant reduction of pressure parameters	[19]
Prospective studies	8821 middle-aged and older men and women	Habitual citrus fruits consumed	Reduction of hypertension incidence	[21]
Cohort clinical trial	10,000 Polish subjects	Habitual consumption of flavonoids, among which flavanones	Reduction of incidence of metabolic syndrome	[22]
Clinical trial of 5 weeks	12 mild hypertension (stage I) subjects	Sweetie fruits (containing 25% naringin and 30% narirutin)	Significant reduction of diastolic pressure parameters	[23]
Clinical trial of 4 weeks	24 overweight subjects	Hesperidin (292 mg, corresponding to levels in 500 mL of orange juice)	Pressure parameter reduction (4 mmHg), amelioration of post-prandial microvascular reactivity	[24]
Controlled clinical trials of 3 weeks	28 subjects with metabolic syndrome	Capsules of hesperidin (500 mg/day)	Reduction of sE-selectin expression, cholesterol and ApoB level reduction, enhancement of NO levels	[25,26]
Clinical trials of 6 months	52 post-menopausal women	Intake of grapefruit juice (containing 105 mg of naringenin)	Improvement of arterial stiffness	[27]
French prospective cohort study	59 middle-aged women	Habitual intake of flavonoids, among which flavanones	Improvement of vascular function and slowing down of atherosclerotic progression	[28]
Clinical trials of 2 months	30 healthy subjects + 30 hypercholesterolemic subjects	Capsules of naringin (400 mg/day)	Reduction of LDL-C, cholesterol and ApoB levels. Increase of HDL-C levels and detoxifying enzymes.	[29]
Clinical trial of 4 or 6 months	20 healthy subjects and 33 subjects with metabolic syndrome	Intake of 300 mL of fruit juice (containing 95% of citrus flavonoids)	No variations of glucidic parameters, improvement of lipidic panel	[30]
Clinical trial of 4 weeks treatment	25 hyperchoesterolemic subjects	Intake of 200 mL of blond orange juice (three times a day)	ApoA levels reduction	[31]
Prospective study with 6-month treatment	80 patients with mild hypercholesterolaemia	Intake of Bergavit^®^ (bergamot extract containing 150 mg/day of flavonoids)	Improvement of lipidic panel and reduction of cholesterol levels	[32]
Randomized controlled study of 4 weeks of treatment	204 healthy and with moderate hypercholesterolaemia subjects (men and women)	Intake of capsules containing naringin+hesperidin (500 mg and 800 mg/day respectively)	No improvement of lipidic panel	[33]
Clinical trial of 4 weeks of treatment	24 overweight subjects	Hesperidin (292 mg/day)	No improvement of lipidic panel	[34]
